# Peptide array-based screening reveals a large number of proteins interacting with the ankyrin-repeat domain of the zDHHC17 *S*-acyltransferase

**DOI:** 10.1074/jbc.M117.799650

**Published:** 2017-09-07

**Authors:** Kimon Lemonidis, Ruth MacLeod, George S. Baillie, Luke H. Chamberlain

**Affiliations:** From ‡ The Strathclyde Institute of Pharmacy and Biomedical Sciences, 161 Cathedral Street, University of Strathclyde, Glasgow G4 0RE and; the § Institute of Cardiovascular and Medical Sciences, University of Glasgow, Wolfson Link Building, Glasgow G12 8QQ, Scotland, United Kingdom

**Keywords:** cytoskeleton, enzyme, peptide array, protein palmitoylation, protein-protein interaction, ankyrin-repeat domain, zDABM, zDHHC13, zDHHC17

## Abstract

zDHHC *S*-acyltransferases are enzymes catalyzing protein *S*-acylation, a common post-translational modification on proteins frequently affecting their membrane targeting and trafficking. The ankyrin repeat (AR) domain of zDHHC17 (HIP14) and zDHHC13 (HIP14L) *S*-acyltransferases, which is involved in both substrate recruitment and *S*-acylation-independent functions, was recently shown to bind at least six proteins, by specific recognition of a consensus sequence in them. To further refine the rules governing binding to the AR of zDHHC17, we employed peptide arrays based on zDHHC AR-binding motif (zDABM) sequences of synaptosomal-associated protein 25 (SNAP25) and cysteine string protein α (CSPα). Quantitative comparisons of the binding preferences of 400 peptides allowed us to construct a position-specific scoring matrix (PSSM) for zDHHC17 AR binding, with which we predicted and subsequently validated many putative zDHHC17 interactors. We identified 95 human zDABM sequences with unexpected versatility in amino acid usage; these sequences were distributed among 90 proteins, of which 62 have not been previously implicated in zDHHC17/13 binding. These zDABM-containing proteins included all family members of the SNAP25, sprouty, cornifelin, ankyrin, and SLAIN-motif containing families; seven endogenous Gag polyproteins sharing the same binding sequence; and several proteins involved in cytoskeletal organization, cell communication, and regulation of signaling. A dozen of the zDABM-containing proteins had more than one zDABM sequence, whereas isoform-specific binding to the AR of zDHHC17 was identified for the Ena/VASP-like protein. The large number of zDABM sequences within the human proteome suggests that zDHHC17 may be an interaction hub regulating many cellular processes.

## Introduction

Protein *S*-acylation, the reversible attachment of fatty acids onto cysteines, is a widespread post-translational modification. The regulatory effects of *S*-acylation are protein-specific but this modification typically impacts membrane binding, intracellular trafficking, protein stability, and protein-protein interactions (1–4).

*S*-Acylation reactions are mediated by the large (24 isoforms in humans) zDHHC enzyme family. Collectively, these enzymes are responsible for the modification of up to 10% of the proteome (5). However, we currently lack a detailed understanding of substrate specificity in the zDHHC family, since knowledge about the enzymes that modify specific proteins is sparse.

The zDHHC13 and zDHHC17 enzyme isoforms contain N-terminal ankyrin repeat (AR)^3^ domains. AR domains are typically involved in protein-protein interactions, and indeed our previous work (6, 7), and that of others (8–11), demonstrated that the AR domains of these zDHHC enzymes are involved in both substrate binding as well as *S*-acylation-independent functions.

Efforts to profile zDHHC enzyme substrates and binding partners have had limited success to date, with proteomics analysis of zDHHC knock-out samples only revealing a small number of high-confidence targets (12, 13). This might reflect the use of gene-trap models with residual zDHHC expression, or the partial overlap in the zDHHC isoforms that can modify a specific protein. To examine zDHHC-substrate specificity and interactions, we have adopted a different strategy that involves the identification of a “consensus” recognition motif for the AR domains of zDHHC17 and zDHHC13. We previously showed that a (VIAP)(VIT)*XX*QP motif present in several targets of zDHHC17/13 mediates interaction with these enzymes (7). To capitalize on this novel observation, we have now undertaken peptide array mapping to generate a set of sequence rules for zDHHC17 binding. These sequence rules have allowed the prediction and validation of binding sites in a wide range of cell proteins. We now term this sequence motif as zDABM (zDHHC-AR-binding motif).

## Results

### Establishment of sequence rules governing binding of peptides to the AR of zDHHC17

**Figure 1.**
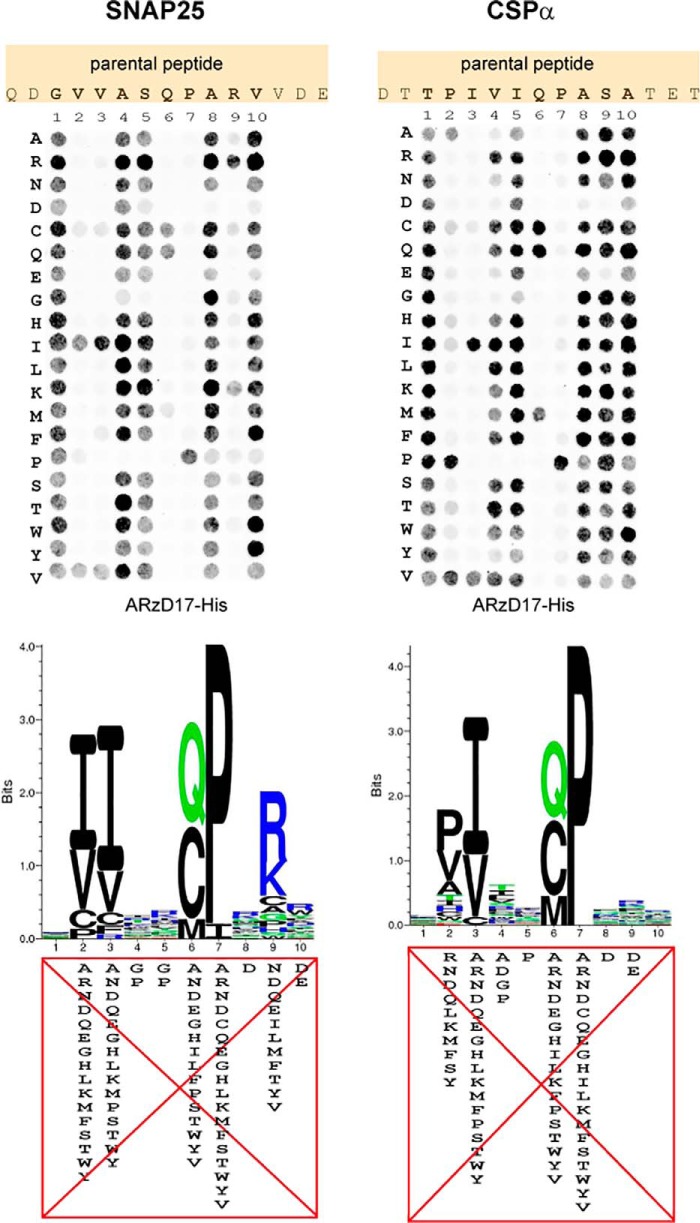
**Assessment of amino acid preferences for binding to the AR domain of zDHHC17.** SNAP25 and CSPα 15-mer peptides having all possible amino acid substitutions within a 10-amino acid region were constructed on peptide arrays. The ability of each peptide to interact with the AR of zDHHC17 was assessed by far-Western blotting, using a histidine-tagged purified human AR domain of zDHHC17 (ARzD17-His; 51–288 amino acids), and detection using a histidine tag antibody. Corresponding sequence logos showing preferences for each amino acid within these 10-amino acid stretches were subsequently created. Signals for each peptide spot were quantified and normalized against the average signal of wild-type peptides for each protein. Peptides having signal intensities less than 5% of wild-type peptides were considered non-binders and were penalized with a score of zero; the rest were expressed as frequencies (normalized so that the sum of scores for each position equals to 1). The derived PSSMs (see supplemental Fig. S1*A*) were used for the generation of sequence logos (generated by Seq2Logo; Shannon type with no clustering method). The highly disfavorable amino acid substitutions (with scores of less than 0.005) for each position are shown.

Peptide arrays consisting of 15-mers of SNAP25 and CSPα peptides were constructed, with all amino acids within a 10-amino acid region containing the zDABM serially substituted to any possible amino acid. Binding of purified His_6_-tagged AR domain (residues 51–288) of zDHHC17 (ARzD17-His) to each of these peptides was subsequently assessed by far-Western blotting using a histidine tag antibody (Fig. 1). Binding of ARzD17-His was more sensitive to alterations of amino acids at positions 2–3 and 6–7 for both SNAP25 and CSPα peptides; however, amino acid preferences at position 2 for the ARzD17-His binding were slightly different for SNAP25 and CSPα (*e.g.* preference for proline in CSPα), whereas additional sensitivity at position 9 was observed for SNAP25 peptides (Fig. 1). Interestingly, cysteine and methionine could compensate well for the loss of glutamine at position 6 in both SNAP25 and CSPα. Additionally, as derived from both SNAP25 and CSPα arrays, no amino acid preference for ARzD17-binding was observed at position 1; amino acids glycine and proline were strongly disfavored at positions 4 and 5; and negatively charged (Asp and Glu) amino acids were disfavored at positions 8–10.

### Prediction of zDABM sequences within the human proteome

**Figure 2.**
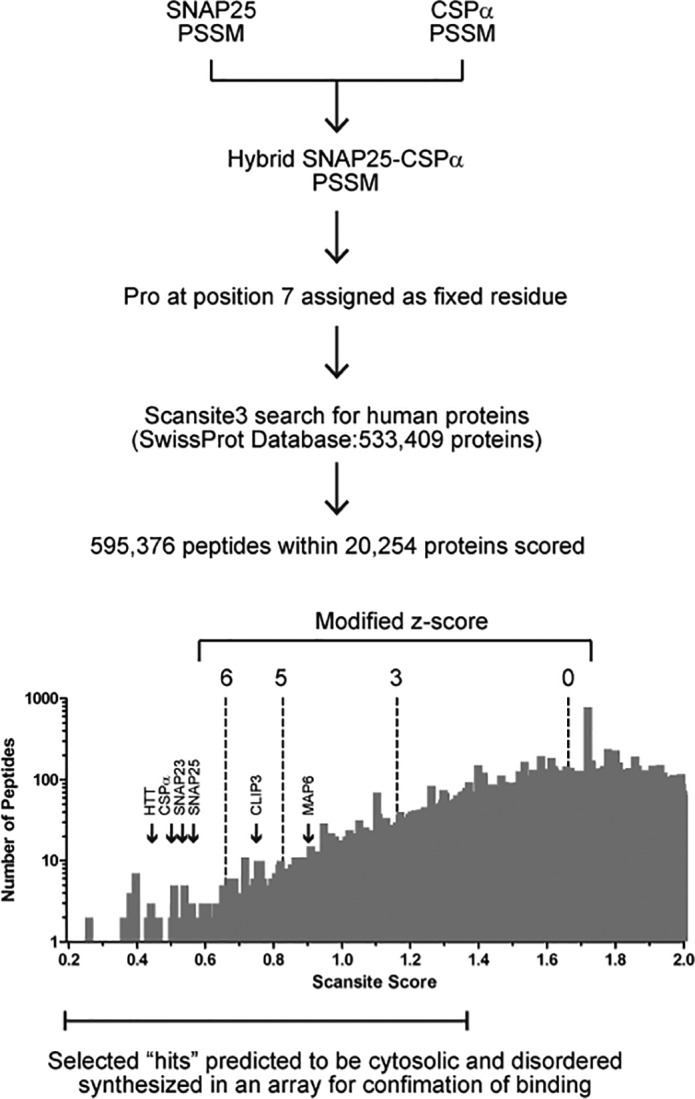
**Procedure used for the *in silico* prediction of zDHHC-AR-binding motif (zDABM) sequences across the human proteome.** A hybrid SNAP25 and CSPα PSSM was created, from averaging scores from individual SNAP25 and CSPα PSSMs (derived from quantification of far-Western blots shown in Fig. 1) and adjusting this PSSM for Scansite, with Pro at position 7 assigned as a fixed amino acid (see ”Experimental procedures“ for details). The derived PSSM was used for matching peptides across the human proteome (SwissProt database; UniProt release 2011_11). The number of peptides scored and the distribution of peptides for each score are shown. Selected hits with Scansite scores ranging between 0.2 and 1.4 (see ”Experimental procedures“ for more details) were filtered for cytosolic localization and disorder prediction to be included for validation of ARzD17 binding. Peptides with a modified z-score (number of S.D. below the median Scansite score −1.663) above 6 were considered as high confidence hits. The positions in the histogram of the six peptides that have been previously shown to bind to the AR of zDHHC17 and zDHHC13 are shown.

To incorporate information derived from both peptide arrays, position-specific scoring matrices (PSSMs) were created for both arrays, based on quantification of far-Western spots, and a hybrid PSSM was derived from the two matrices; this PSSM was adjusted to be used as input motif for the search of human protein sequences that are good matches for this matrix, using Scansite 3 (supplemental Fig. S1*B*). A total of 533,409 peptides were scored (Scansite scores ranging from 0.1931 to 2), with only a minority of peptides occupying lower (and thus best matching) scores (Fig. 2 and supplemental Table S1). Modified z-scores (number of S.D., below the median Scansite score) were also calculated for each scored peptide. Peptides having modified z-scores above 6 (Scansite scores below 0.6601) were considered as high confidence hits; both SNAP25 and CSPα, as well as Huntingtin (HTT) and SNAP23, from the previously identified zDABM sequences (7), were scored within this range, which corresponds to only the top 0.11% percentile of the scored peptides; medium and low confidence levels were arbitrary chosen to include peptides having modified z-scores between 5 and 6, and between 3 and 5, respectively; within these three confidence levels all six previously reported zDABM sequences (7) are scored as good matches for ARzD17-binding (Fig. 2), and this range accounts for the top 5% percentile of the scored peptides.

### Validation of putative zDABM sequences for binding to the AR domain of zDHHC17

More than 2,600 sequences deriving from the Scansite search were analyzed for cytosolic localization and disorder (for more information see ”Experimental procedures“ and supplemental Table S1). We identified a total of 590 disordered and cytosol-localized sequences, which were distributed among 224 proteins (supplemental Table S2). 107 of these peptides (distributed in 96 proteins) were chosen for assessment of binding to ARzD17-His; these included 51 high confidence sequences, 28 medium confidence sequences, 26 low confidence sequences, and 2 sequences below the lowest confidence threshold level. 40 of the 107 sequences derived from proteins are either known or are speculated to be zDHHC17 interactors. One sequence from the nuclear MAX gene-associated protein (MGAP), for which no cytosolic localization has been documented, was also included in peptide synthesis to serve as positive control, because this was at the top 0.01% percentile, with a Scansite score below 0.4 and a modified z-score above 7.5. Additionally, two non-natural peptides were synthesized: one with a highly favorable ARzD17-binding amino acid sequence, and another one with a highly disfavorable amino acid sequence (according to the SNAP25-CSPα PSSM); these two peptides, served as positive and negative controls, respectively.

**Figure 3.**
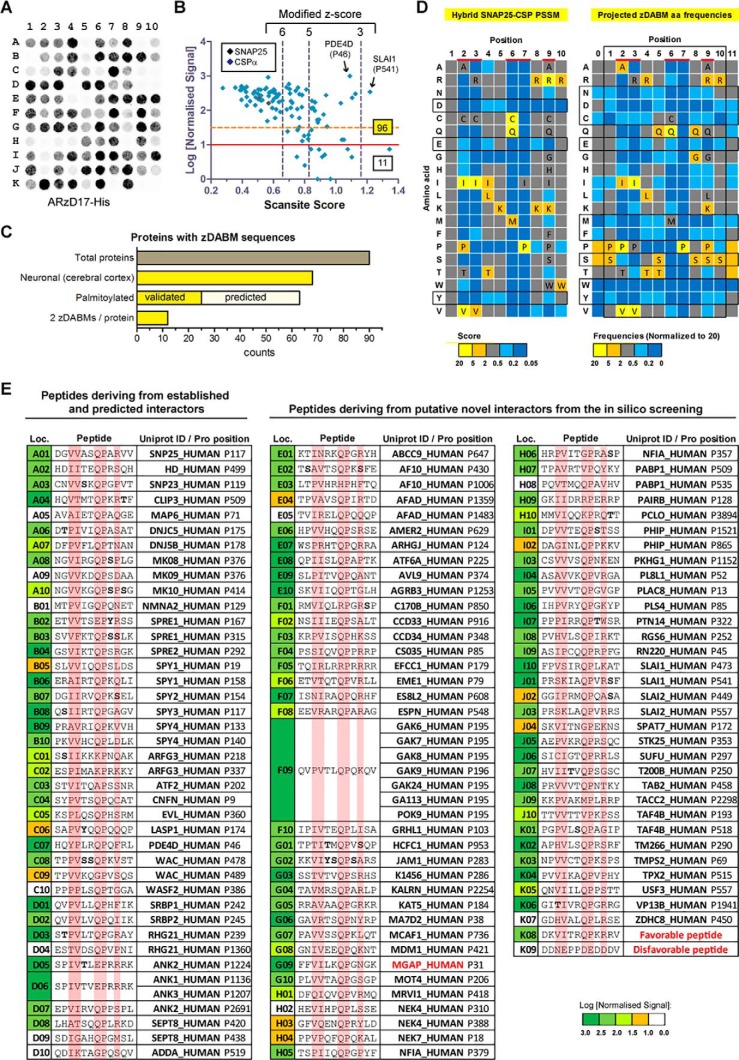
**Validation of predicted zDABM sequences.** A peptide array was constructed comprising a total of 109 12-mer peptides, which correspond to 107 human sequences, and two artificial sequences: one expected to bind ARzD17 (favorable peptide) and one not expected to bind (disfavorable peptide). Binding to ARzD17 was assessed by far-Western blotting using purified human ARzD17-His (51–288 amino acids) and detection with a histidine tag antibody. Quantified signals were normalized to the highest observed signal, which was given an arbitrary value of 1000; peptides with values less than 10 were considered non-binders. *A,* peptide array far-Western blot. *B,* normalized signal intensities were plotted against the Scansite-derived score. Peptides *below the red line* (log values <1) were considered as non-binders; whereas peptides *below the dashed orange line* (log values <1.5) were considered as weak binders. The total numbers of binding and non-binding peptides, as well as the position of SNAP25 and CSPα in the plot, are shown. *C,* bar graph showing the total number of proteins containing zDABM sequences and the number that are known to be neuronal, palmitoylated (SwissPalm database), or predicted to be palmitoylated (palmitoylation sites were predicted by CSS Palm 3.0 using high threshold setting), as well as the number of proteins that contain two zDABM-binding sites as opposed to one site (the nuclear protein MGAP was excluded from these analyses). *D,* comparison between the amino acid preferences defined by the hybrid SNAP25-CSPα PSSM, and the amino acid preferences defined by the projected amino acid frequency for each position of zDABM-binders. For the projected amino acid frequencies, the remaining high-confidence sequences were also considered on top of all the natural peptides identified as zDABM sequences. The more critical binding positions are highlighted in *red*, with all but disfavorable amino acids for these positions shown; favorable-only amino acids for all other positions are indicated too. Amino acids within the motif that appear highly disfavorable (in at least 7/10 positions) or favorable (in at least 5/10 positions) are highlighted in a *box. E,* peptides used at the corresponding locations on the peptide array, with the most critical for binding positions highlighted; corresponding peptide positions for each peptide within human proteins are shown. Residues that are known to be modified by phosphorylation (PhosphoSite database) are indicated in *bold*. Non-natural zDHHC17-interactors (proteins that are only known to be nuclear and artificial peptide sequences) are shown in *red*.

12-mer peptides of the 109 sequences mentioned above (Fig. 3*E*) were synthesized in an array, and their ability to bind ARzD17 was assessed by far-Western blot analysis, using purified ARzD17-His and detection with histidine tag antibody (Fig. 3*A*). A total of 96 natural occurring zDABM sequences were identified. As expected, the artificial favorable peptide was a strong binder, whereas binding of the artificial disfavorable peptide to ARzD17 was hardly detected. Surprisingly, the zDABM sequence of MAP6, although previously found to be involved in interaction with the AR domain of zDHHC17/13 (7), was not sufficient to promote ARzD17 interaction on its own (Fig. 3*A*), which suggests that MAP6 may require a sequence greater than 12 amino acids, or additional domains for stable interaction with zDHHC17. All high confidence hits tested (51 sequences with z-scores above 6, including MGAP) were able to bind ARzD17 quite strongly, whereas at lower threshold levels, a number of weak binders or no-binders appeared, along with the strong binders (Fig. 3*B*).

In our effort to identify which sequences of a given protein may be involved in ARzD17-binding, we included (in our 109 peptide array) two different peptides for the same protein, for 17 of the proteins screened. To our surprise, in 12 of the 17 proteins tested, both peptides could interact with ARzD17 (Fig. 3*C*). Most of the proteins with confirmed zDABM sequences are known to be expressed in neuronal cells, and are either known, or predicted to be palmitoylated (Fig. 3*C* and supplemental Table S2).

To get a good approximation of amino acid preferences for ARzD17 binding within naturally occurring sequences, occurrences of each amino acid at each position were calculated within all zDABM sequences, including: both verified for ARzD17-binding protein sequences, as well as the rest of the 66 high-confidence hits (which although not verified for binding, are expected to be true zDABM sequences); the inclusion of the latter group in the analysis was done to compensate for any bias introduced in the selection of peptides to be verified as zDABM sequences. The resulting amino acid frequencies for each position reveal an unexpected versatility in amino acid usage for ARzD17-binding, as well as marked absence of particular amino acids (such as Glu, Asp, Asn, Cys, Phe, Met, Trp, and Tyr) in all positions (Fig. 3*D*). Serine, on the other hand was the most favorable amino acid, but in positions less critical for binding. Putative phosphorylation of these serine residues is expected to negatively affect ARzD17-binding, because no negatively charged residues (Asp and Glu) are favored for binding at equivalent positions. Serine, threonine, and tyrosine residues within zDABM sequences, whose phosphorylation has been confirmed, are shown in Fig. 3*E*.

### zDABM sequences are enriched in proteins involved in cytoskeletal organization and cell communication, and in certain protein families

**Figure 4.**
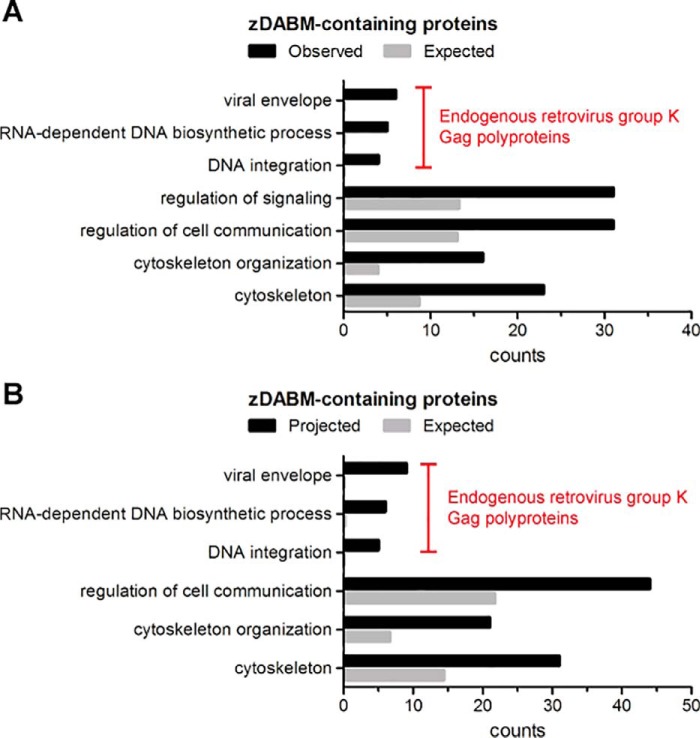
**Gene Ontology (GO) biological processes and cellular components enriched within proteins possessing zDABM sequences.** *A,* the 89 genes corresponding to the 90 proteins with zDABM sequences were analyzed for enrichment of biological processes and cellular components using the PANTHER Classification system. GO processes/components that were enriched by 2-fold or more and were deemed statistically significant (*p* < 0.05) after Bonferroni correction, are indicated, along with the proteins counts for each GO entry. Enriched parental GO terms with the same observed proteins are not shown. *B,* the same analysis was performed as in *A*, but this included the 66 genes corresponding to proteins having high confidence for zDHHC17-binding sequences (modified z-scores above 6) as well, but whose corresponding peptides were not verified for ARzD17-binding.

To gain insight into the functions that may be regulated by zDABM-binding, we assessed whether there is enrichment for certain biological processes or cellular components within the zDABM-containing proteins. The 89 genes corresponding to the 90 proteins with zDABM sequences identified (GAK9 and POK9 derive from the same gene) were thus included for such analysis, using the PANTHER Classification system and Bonferonni correction; this analysis revealed that endogenously derived zDABM-containing proteins are enriched in cytoskeleton, and in functions involved in cytoskeletal organization, and regulation of signaling and cell communication (Fig. 4*A*). Due to the inclusion of 6 retrovirus-derived Gag polyproteins (which share the same zDABM sequence) in our dataset, significant enrichment for viral envelope localization and viral processes (RNA-dependent DNA biosynthesis and DNA integration) were also observed (Fig. 4*A*). To compensate for any bias that may have been introduced by the selection of peptides (and thus the proteins) to be validated for ARzD17 binding, we repeated the analysis, including the 66 extra proteins for which high confidence for ARzD17-binding sequences were found, but whose sequences were not verified for binding. The only enrichment that was lost by the inclusion of these proteins was that for regulation of signaling (Fig. 4*B*).

We next searched whether zDABM sequences are enriched in certain protein families. Indeed, we found that, apart from the SNAP25 family whose two members (SNAP25 and SNAP23) have already being confirmed to have zDABM sequences (7), there were additionally 4 protein families with all members represented in zDABM-containing proteins; these were: the Sprouty (SPY1–4), Cornifelin (CNFN, PLAC8, and PL8L1), Ankyrin (ANK1–3), and SLAIN-motif (SLAI1–2) families. Additionally, zDABM sequences were also found in 2 SPRED (Sprouty-related, EVH1-domain containing) proteins (SPRE1–2), which, like the Sprouty proteins, also have a (putatively highly palmitoylated) C-terminal cysteine-rich Sprouty domain.

### zDABM-containing proteins are able to bind to the AR of zDHHC17 due to their zDABM sequence

**Figure 5.**
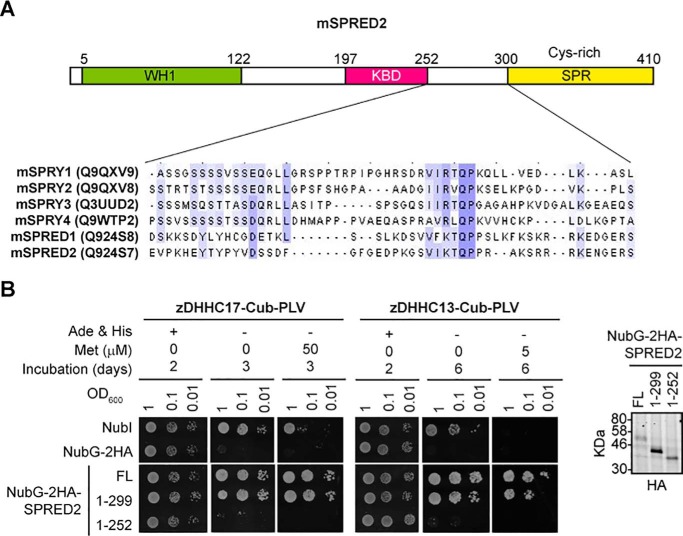
**A homologous region among all Sprouty proteins and SPRED1–2, which is located proximal to the Sprouty domain, is required for SPRED2-zDHHC17/13 interaction.** *A,* domain architecture of murine SPRED2 showing the position of the homologous zDHHC17-binding sequence shared among all Sprouty proteins and SPRED1–2 (*WH1*, WASP-Homology 1; *KBD*, c-Kit-binding domain; *SPR*, Sprouty domain). Mouse SPRY1–4 and SPRED1–2 proteins (UniProt entries shown in parentheses) were aligned using Clustal Omega, and the alignment corresponding to 252–300 amino acids of SPRED2 is shown, with *blue-shaded areas* indicating identical amino acids in 3 or more proteins. *B,* interaction of wild-type murine SPRED2, and two C-terminal truncations, with murine zDHHC17 and zDHHC13, as assessed by the mating based SUS. In this system, ubiquitin is split in half, with the N-terminal half (*Nub*) attached to a prey protein (SPRED2 here), whereas the C-terminal half (*Cub*) is fused between a membrane-bound bait protein (zDHHC17 or zDHHC13 here) and the PLV (Protein A, LexA, VP16 fusion) transcription factor. Matings co-expressing zDHHC17/13 and SPRED2 are able to grow in media lacking adenine and histidine only upon the zDHHC-SPRED2 interaction, because the latter will lead to the reassembly of ubiquitin, its recognition by ubiquitin-specific proteases, and the subsequent cleavage and translocation of the PLV transcription factor to the nucleus, where it can activate genes essential for adenine and histidine synthesis. The I13G substitution on Nub (NubG *versus* NubI) prevents spontaneous reassembly of the two ubiquitin halves in the absence of interaction. Protein levels of NubG-2HA-SPRED2 constructs (shown on the *right*) were assessed by an HA antibody.

We next assessed whether full-length zDABM-containing proteins can interact with the AR domain of zDHHC17 (and zDHHC13), and whether such interactions are truly dependent on the zDABM sequence. As mentioned above, all Sprouty and SPRED proteins have a conserved C-terminal Sprouty (SPR) domain. To assess whether this domain or a zDABM sequence is involved in zDHHC17 interaction, we utilized both full-length SPRED2, as well as two truncation mutants lacking, either the SPR domain, or both the SPR and a region containing the zDABM sequence (SPRED2 has only one zDABM sequence). These constructs were assessed for interaction with both zDHHC17 and zDHHC13 (which we previously showed to interact with zDABM too) using the mating-based split-ubiquitin system (SUS) in yeast. As shown in Fig. 5*A*, all Sprouty proteins and SPRED1–2 have a homologous zDABM sequence proximal to their conserved C-terminal SPR domain. Our SUS assay with the above SPRED2 constructs revealed that both zDHHC17 and zDHHC13 can interact with SPRED2, and that this interaction is not dependent on the SPR domain, but on the region containing the zDABM sequence (Fig. 5*B*).

**Figure 6.**
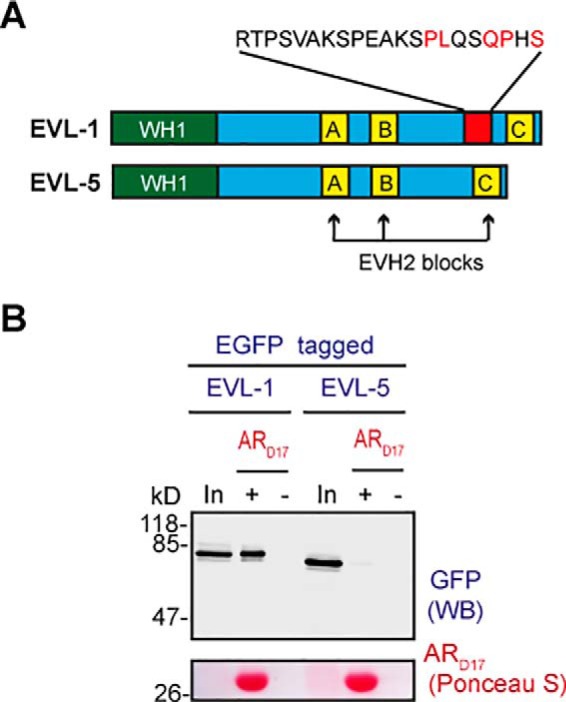
**A novel EVL isoform lacking the zDABM sequence is unable to interact with the AR domain of zDHHC17.** *A,* domain architecture of EVL isoforms 1 and 5 (*WH1*, WASP-homology 1; *EVH2*, Ena/VASP-homology 2). Isoform 5 is missing a 21-amino acid region (shown in *red*) containing the zDABM sequence (amino acid sequence of this stretch is shown with the critical for ARzD17-binding positions indicated in *red*). *B,* EGFP-tagged EVL-1 and EVL-5 human proteins were expressed in HEK293T cells, and their ability to interact with the AR domain of zDHHC17 was assessed by pull downs of corresponding lysates by ARzD17-His (51–304 amino acids of human zDHHC17). 7.5% of total inputs and 40% of total bound fractions were run on 12% gels, and following transfer, blots were stained by Ponceau S solution and probed with a GFP antibody.

Ena/VASP-like protein (EVL) is an actin-associated protein, recently identified to interact with zDHHC17 in a yeast two-hybrid system (11). Like SPRED proteins, it also contains an N-terminal WH1 domain, whereas its single zDABM sequence contains at position 3 the disfavorable ARzD17-binding amino acid leucine. In our attempt to clone isoform 1 of EVL for assessment of zDHHC17-binding, we also isolated a novel isoform (isoform 5), which is lacking a 21-amino acid stretch containing the EVL zDABM sequence (Fig. 6*A*); the cDNA sequence of this isoform has been submitted to GenBank^TM^ (accession number KY819016), and its translation product corresponds to EVL isoform 5 (Q9UI08-5 entry in UniProt). ARzD17-His pull downs of HEK293T lysates expressing these two EGFP-EVL isoforms revealed that only EVL isoform 1 (EVL-1), which has the zDABM sequence, can bind to the AR domain of zDHHC17, whereas EVL isoform 5 (EVL-5), which lacks a zDABM, cannot (Fig. 6*B*).

### PAIR-BP1 and SLAIN1 are able to interact with the zDHHC17 despite possessing non-favorable amino acids within their zDABM sequences

**Figure 7.**
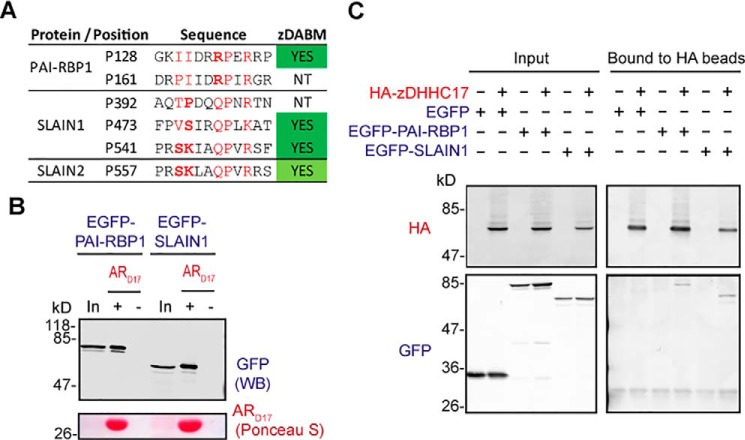
**PAI-RBP1 and SLAIN1 are able to bind to zDHHC17, despite possessing disfavorable amino acids within their zDABM sequences.** *A,* confirmed and speculated zDABM sequences of PAI-RBP1, SLAIN1, and SLAIN2 are shown, with amino acids located at critical binding positions shown in *red* and disfavorable amino acids (according to SNAP25 and CSPα PSSMs) indicated in *bold* (*NT*, not tested). These zDABM sequences exist in all known isoforms of these proteins. The proline position corresponds to the location of the zDABM in the canonical sequence for each protein. *B,* EGFP-tagged PAI-RBP1 and SLAIN1 human proteins (isoforms 3 and 2, respectively) were expressed in HEK293T cells, and their ability to interact with the AR domain of zDHHC17 was assessed by pull downs of corresponding lysates by ARzD17-His (51–304 amino acids of human zDHHC17). 7.5% of total inputs and 40% of total bound fractions were run on 12% gels, and following transfer, blots were stained by Ponceau S solution and probed with a GFP antibody. *C,* lysates of HEK293T cells co-expressing HA-zDHHC17 and EGFP, EGFP-tagged PAI-RBP1 (isoform 2), or EGFP-tagged SLAIN1 (isoform 3) were subjected to co-immunoprecipitation using HA beads. 6% of the total inputs and 25% of the immunoprecipitated fractions were resolved on 10% polyacrylamide gels and then visualized by Western blotting using HA and GFP antibodies.

Because a number of peptides with non-favorable amino acids at critical for binding positions came up as ARzD17-binders, we were particularly interested in assessing if proteins containing them are able to interact with zDHHC17 as well. Thus, apart from EVL isoforms 1 and 5, we also cloned and subsequently expressed in HEK293T cells EGFP-tagged versions of human PAI-RBP1 (plasminogen activator inhibitor 1 RNA-binding protein) and SLAIN1 (SLAIN motif-containing protein 1). PAI-RBP1 presumably utilizes 2 different sequences for ARzD17-binding, and both of them have arginine instead of glutamine at position 7; SLAIN1 on the other hand, may use 2–3 different sequences for ARzD17-binding, each having unfavorable amino acids at positions 2–3 (Fig. 7*A*). All isoforms of these proteins contain the above zDABM sequences, including the shorter versions used in this experiment (isoform 2 of SLAIN1 and isoform 3 of PAIBP1). ARzD17-His pull downs of HEK293T lysates expressing the above proteins revealed that both PAI-RBP1 and SLAIN1 can bind to the AR domain of zDHHC17 (Fig. 7*B*), whereas co-immunoprecipitation assays showed that these proteins also form complexes with full-length zDHHC17 (Fig. 7*C*).

## Discussion

In this study we have evaluated binding of a large number of peptides (based on amino acid sequences of SNAP25 and CSPα) to the AR domain of zDHHC17. The binding profile of this comprehensive set of peptides enabled us, not only to construct a sequence rule-based consensus for ARzD17 binding, but also to search for and subsequently validate a large number of human sequences as zDABM-bearing sequences. The striking preference for specific amino acids at positions 2–3 (aliphatic ones) and 6–7 (QP dipeptide) within zDABM sequences observed before (7) has been confirmed here (Fig. 1). Indeed a very recent structural study (Ref. 14; published while this paper was under review) showed that the above residues in SNAP25 zDABM form a number of important contacts with a region between ankyrin repeats 1 and 3 of zDHHC17; more specifically, interactions between, Val-113 and Pro-117 of the analyzed SNAP25 peptide, and Asn-100 and Trp-130, respectively, of zDHHC17, were found to be particularly important. Nevertheless, despite the striking preference for particular amino acids in these positions, a large number of peptides having non-favorable amino acids at those positions are still able to bind strongly to ARzD17. The favoritism for certain amino acids at various positions thus seems to be influenced by the surrounding amino acids within the zDABM sequence: for example, the amino acid sequence of the SNAP25 zDABM has a strong preference for lysine or arginine at position 9, whereas the CSPα zDABM permits a wider variety of residues at this position; the CSPα zDABM also strongly favors proline and alanine at position 2, which are either less favored or disfavored for ARzD17-binding, respectively, in the SNAP25 sequence; similarly, the disfavored dipeptide sequences at positions 2–3, YP and SK, appear to be permitted/favored in the zDABM sequences of PDE4D (Pro-46) and SLAIN1 (Pro-541), respectively. zDABM sequences thus appear very versatile in the usage of amino acids.

Although the sequence assessment of non-binders and binders could hardly indicate amino acids that are absolutely detrimental for ARzD17-binding, the binding pattern of the over 500 peptides tested in our study reveals marked preferences for particular amino acids at certain positions, and at the same time strong lack of preference for other amino acids (Asn, Asp, Glu, Cys, Phe, Met, Trp, and Tyr) at all positions tested; the latter partly reflects the propensity of certain amino acids (Cys, Phe, Met, Trp, and Tyr) not to be present in disordered regions (15). The strong preference for positively charged residues (Arg and Lys) over negatively charged ones (Asp and Glu) at certain positions (4 and 9), as well as the general lack of preference of negatively charged residues, indicates the possible existence of a negatively charged patch in or around the zDABM-binding pocket in zDHHC17. Indeed, Glu-89 and Asp-122 of zDHHC17, which contribute to the interaction with the zDABM sequence of SNAP25, may provide the negative charge involved in favoring positive over negative charged residues in zDABM sequences (14). Additionally, a strong preference for serine residues at the less critical binding positions was observed within the confirmed and highly predicted sets of zDABM sequences. Putative phosphorylation of these serines is expected to impair binding to the AR domain of zDHHC17/13. A phosphorylation-dependent binding switch-off has been previously documented for the Ankyrin repeat of ANKRA2 and the phosphorylated LYTSP(pS)LPNITL sequence of HDAC4 (16). Unexpectedly, cysteine and methionine were found to compensate reasonably well for the lack of glutamine at position 7. The reason for that is unclear, because Gln-116 of a SNAP25 peptide is involved in hydrogen bonding with Glu-89 and Asp-122 of zDHHC17 (14), and both cysteine and methionine would be unable to substitute these bonds. It is possible that some substitutions force peptides to create new bonds, originally absent in wild-type proteins, and such flexibility in new bond formation was recently shown for Gln-116 of SNAP25 upon zDHHC17 binding (14).

Despite the versatility of amino acid usage at critical binding positions of zDABM sequences, our approach could accurately predict a large number of zDABM sequences, with 100% accuracy (51 tested) for peptides with modified z-scores above 6 (Scansite scores below 0.6601). In total, we were able to identify zDABM sequences in 28 of the known and speculated zDHHC17/13 interactors, and in 62 proteins not previously implicated in zDHHC17/13-binding. Seven of these novel zDHHC17 interactors are retrovirus group K Gag polyproteins sharing the same zDABM sequence. Increased mRNA levels of such proteins have been linked with cancer and a number of autoimmune and neurological diseases (17). Whether zDHHC17-binding can enhance or prevent such processes remains to be found. Other families whose members were enriched among zDABM-containing proteins were: the Sprouty, Ankyrin, Cornifelin, and SLAIN-motif families, whereas 2 members of the Sprouty-related EVH1-domain containing (SPRED) protein family were identified to have zDABM sequences.

Sprouty and SPRED proteins are known to be negative regulators of MAPK signaling and may thus function as tumor suppressors (18, 19), whereas SPRED1 and SPRED2 have been additionally found to be negative regulators of hematopoiesis (20, 21). Because zDHHC17 has an oncogenic potential upon overexpression (22) and is up-regulated in cells overexpressing an essential hematopoiesis transcription factor (23), it is expected that the interaction of zDHHC17 with Sprouty and SPRED proteins would negatively regulate the functions of the latter.

Ankyrins are major scaffolding proteins that coordinate membrane transporters and cell adhesion proteins within plasma membrane compartments (24); these functions depend heavily on Ankyrin palmitoylation, but among all zDHHC *S*-acyltransferases, only zDHHC5 and zDHHC8 were found to be able to mediate Ankyrin 3 palmitoylation (25). Hence, the interaction of zDHHC17 with Ankyrins either serves some currently unknown *S*-acylation-independent functions, or mediates *S*-acylation of proteins attached to Ankyrins.

Cornifelin (CNFN) is a component of the cornified envelop with high expressions in skin, uterus, and cervix (26). This protein was recently found to be palmitoylated on 5 different sites and to be a substrate of zDHHC13 (27); because zDHHC17 has been shown to have greater activity than zDHHC13 for all interacting proteins tested (6, 28, 29), it is expected that zDHHC17 is able to palmitoylate Cornifelin as well. The placenta-specific gene 8 protein (PLAC8) and PLAC8-like protein 1 (PLAC8L1) also belong to the Cornifelin family. Both proteins, like Cornifelin, have four predicted palmitoylation sites (supplemental Table S3) and an N-terminal zDABM sequence. Hence, these are also likely to be palmitoylated by zDHHC17 and zDHHC13. Although PLAC8 seems to have functions important for a range of processes like immunity (30), brown and white fat cell differentiation (31, 32), and autophagy (33), the function for PLAC8L1 is currently unknown.

SLAIN-motif proteins (SLAIN1 and SLAIN2) are enriched in brain and promote microtubule growth by recruiting the microtubule polymerase ch-TOG to microtubule plus-ends (34). Furthermore, loss of function mutations of SLAIN1 (as well as of MAPK8, which was also identified as zDABM-protein in our screening) are associated with intellectual disability (35). How the zDHHC17 interaction with SLAIN-motif proteins regulates these functions is currently unknown.

SLAIN-motif proteins and Ankyrins are only some of the many neuronal zDABM-containing proteins with roles in cytoskeletal organization and regulation of cell communication, respectively, whereas proteins that regulate signaling, like SPRED and Sprouty, are also very common among zDABM proteins. Importantly, these three processes often converge, especially in brain cells, with neurons executing their function by efficiently communicating with other neurons, as well as glial cells, and transmitting various signals either intracellularly or extracellularly via a network of cell adhesion proteins and cytoskeleton-associated proteins. Hence zDHHC17 may be an important regulator of communicative networks in the brain. Indeed, a sharp decrease in zDHHC17 expression in mice results in numerous behavioral and synaptic deficits (36, 37), which get more severe and become incompatible with life, when zDHHC17 is completely lost (38). These deficits are associated with striatal dysfunction, astrogliosis, and microgliosis (38). The regulation of actin- and microtubule-based processes by zDHHC17 in neurons is probably reflected by the fact that, loss of zDHHC17 in zebrafish, neuronal stem cells, or PC12 cells causes defects in axonal outgrowth (39). The actin-associated EVL protein is a recently identified zDHHC17 interactor (11), which is involved, along with other Ena/VASP proteins, in neuritogenesis and axon formation (40). Rat cortical neurons and glial cells contain two alternatively spliced isoforms of EVL differing in a 21-amino acid sequence (41), whereas similar alternatively spliced products are also reported in frogs (42). Here, we show that alternatively spliced products differing in this sequence exist in humans too, and that the AR of zDHHC17 can only bind EVL when this extra sequence is present; thus EVL binding to zDHHC17 can serve functions associated only with the longer EVL isoform. The latter isoform has been reported to be phosphorylated by protein kinase D on this extra sequence (43), but distal to the zDABM sequence. The exact role of the modification is not known; however, the phosphorylated EVL longer isoform was found to be enriched in tight cell junctions (43).

Unexpectedly, a zDABM-containing peptide of the microtubule-associated protein 6 (MAP6) was not sufficient for binding to the AR domain of zDHHC17. This sequence is known to be implicated in ARzD17/13-binding, because alanine substitution of the conserved proline does impair binding (7). Hence, the zDHHC17/13-MAP6 interaction may require either a longer MAP6-peptide sequence than typically required for zDABMs, or a second interface to stabilize the interaction. It is currently established that structures comprised of 4–6 ankyrin repeats can support binding of up to 8-amino acid peptide stretches (16, 44–46), whereas considerably longer peptide sequences of sodium channel Nav1.2 and Neurofascin peptides (28 and 95 amino acid peptides, respectively) are required for efficient interaction with the 24 ankyrin repeats of Ankyrin proteins (47). Thus the 7 ankyrin repeats of zDHHC17 and zDHHC13 AR domains may be able to accommodate slightly longer than 8-amino acid long sequences, with the exact length being dependent on the individual sequence of each protein. In the case of SNAP25, a longer than 10-amino acid sequence seems to be required for maximal zDHHC17-binding, because a 10-amino acid stretch of SNAP25 could not support a strong interaction with the ARzD17, as full-length SNAP25 could (7, 14). Additionally, the analysis of amino acid frequencies among zDABM-containing proteins has been revealed as disfavorable for ARzD17-binding amino acids in the whole 12-amino acid stretch assessed (Fig. 3*D*), which indicates that many proteins may need sequences of 12 amino acids or longer for efficient ARzD17-binding.

In this work we found that the AR of zDHHC17 could interact with two different sequences located in the same protein, for at least 12 of the 17 proteins tested. Thus a greater number of zDHHC17 binders with two zDABM sequences may potentially exist. Moreover, given that more than 10 putative zDABM sequences per protein were found for certain proteins (supplemental Table S2), it is possible that zDHHC17 may recognize more than 2 sequences within these and other proteins. The above indicate that the AR domain of zDHHC17 (and potentially of zDHHC13) has the capacity to engage with a given number of proteins in different ways, thus forming different complexes according to the way that each protein is presented to its AR domain. This means that blockage of zDHHC17 binding on one site of a protein (*i.e.* by phosphorylation or binding to another protein) may result on this protein being bound to zDHHC17 using a different site; this may subsequently result in a different function executed due to this alternative binding. The ability of zDHHC17 to bind such a large number of sequences with relaxed rules for binding may thus enable this enzyme to bind a plethora of proteins under different orientations; zDHHC17 may thus be a hub for many interactions responsible for dynamic regulation of a multitude of processes.

## Experimental procedures

### Chemicals and antibodies

Unless otherwise stated, all chemicals were purchased from Sigma (Dorset, UK). Mouse His_6_ tag antibody (ab18184) was from Abcam (Cambridge, UK), rat HA from Roche Applied Science (Sussex, UK), and mouse GFP (JL8) from Clontech. Secondary IRDye mouse antibodies were from LI-COR (Cambridge, UK).

### Cloning, mutagenesis, and protein purification

With the exception of human ARzD17-His protein (His-tagged ankyrin-repeat domain of zDHHC17), which is described below, and murine zDHHC17 in HA-pEF-BOS, which was kindly provided by Masaki Fukata (48), all cDNAs were cloned by Gateway technology (6), using the manufacturer's kits and instructions (Life Technologies, Paisley, UK). PCR and site-directed mutagenesis reactions for insertion of STOP codons were performed using a KOD hot-start polymerase kit according to the manufacturer's guidelines (Merck Millipore, Watford, UK). Primers for introducing Gateway compatible adapters by PCR (attB-PCR) were purchased from Life Technologies, whereas primers for site-directed mutagenesis, or PCR amplification with restriction adapters, were purchased from Sigma. All site-directed mutagenesis reactions occurred in entry clones and were confirmed by sequencing (GATC service, Constance, Germany). Original cDNAs used for cloning were as follows: human zDHHC17 was provided by Michael Hayden (49), murine zDHHC17 and zDHHC13 (DHHC22) was provided by Masaki Fukata (48), murine SPRED2 was provided by Akihiko Yoshimura (50), whereas human SLAIN1 (isoform 2), PAI-RBP1 (isoform 3), and EVL (isoforms 1 and 5) were isolated from a human embryonic cDNA brain library. Although isoform 2 of SLAIN1 and isoform 3 of PAI-RBP1 are lacking sequences present in the canonical isoforms of these proteins, they both have all the zDABM sequences predicted in the canonical isoforms.

For the production of pET303-ARzD17-His plasmid, a human zDHHC17 cDNA fragment (encoding for either 51–288 or 51–304 amino acids) was amplified by PCR using appropriate primers containing also the XbaI and XhoI restriction sites, and the resulting PCR product was subcloned into pET303CT-His plasmid (Life Technologies), following XbaI and XhoI digestion. The resulting protein (51–288 or 51–304 amino acids of human zDHHC17, followed by a Leu-Glu linker and a His_6_ tag) was purified from transformed BL21(DE3)pLysS bacterial cells (Life Technologies), as described previously for murine His_6_-AR_D17_ (7). Protein concentration was estimated by Nanodrop 2000c measurement.

### Far-Western blots

Peptide libraries were produced by automatic SPOT synthesis (51). They were synthesized on continuous cellulose membrane supports on Whatman 50 cellulose membranes using Fmoc (9-fluorenylmethyloxycarbonyl) chemistry with the AutoSpot-Robot ASS 222 (Intavis Bioanalytical Instruments, Berlin) (52). The SNAP25 and CSPα arrays consisted of 15-mer peptides (200 peptides each), with all possible amino acid substitutions within a 10-amino acid stretch (sequences and serial substitution shown in Fig. 1). The dried membranes were submerged in 100% ethanol for 2 min, washed briefly with distilled water, and then incubated with blocking buffer: 5% (w/v) milk in PBS-T (PBS containing 0.02% Tween) for 2 h at room temperature. Then, after a brief washing with PBS-T, overnight incubation with the ARzD17-His protein (500 nm in PBS-T) took place at 4 °C. Membranes were then washed extensively with PBS-T, incubated with mouse His_6_ tag antibody (1/2,000 dilution in PBS-T) for 1 h at room temperature, washed again with PBS-T, incubated with mouse secondary antibody (1/10,000 dilution in PBS-T), and after extensive washes with PBS-T containing 0.2% Tween, ARzD17-His-bound peptides were visualized using a LI-COR infrared scanner. Spots were quantified using Image Studio software (version 2.0), with an area of the blot containing no peptide assigned as background.

### PSSMs

Derived signals from quantification were normalized against the average signal intensity of wild-type peptides for either SNAP25 or CSPα. Peptides displaying signals that were less than 5% of average wild-type intensities were considered non-binders and were thus given a score of: (*a*) zero for PSSMs to be used for generation of sequence logos, or (*b*) a score of 0.05 for PSSMs intended to be used for Scansite. The rest of the signals were expressed as frequencies: signals were normalized so that the sum of scores for each peptides equals (*a*) 1 for each position for PSSMs used for sequence logo generation (supplemental Fig. S1*A*), or (*b*) 20 in PSSMs to be used for Scansite search. For Scansite search, a hybrid PSSM was subsequently produced by averaging each of the scores (ranging from 0.05 to 20) from SNAP25 and CSPα PSSMs. Proline at position 7 was then assigned as the fixed residue: the Pro substitution was given a score of 21 in the hybrid PSSM, whereas all other substitutions at that position were given a score equal to zero (supplemental Fig. S1*B*).

### In silico prediction of zDABM human sequences

The hybrid SNAP25-CSPα PSSM (with the Pro assigned as fixed residue at position 7) was selected as the “input motif” in Scansite 3 for the search of human sequences (SwissProt database; 2011_11 release) matching this motif. Modified z-scores (number of S.D. below the median Scansite score) were also calculated for each peptide. Because zDABM sequences are positioned within disordered regions (7), and they need to be cytosolic to physically interact with zDHHC17 (and/or zDHHC13), selected hits were validated for disorder and cytosolic localization; these included all sequences from proteins with modified z-scores above 6, and a number of sequences with modified z-scores above 3, including other sequences from the above proteins, all known and speculated zDHHC17/zDHHC13 interactors, and a few randomly selected peptides/proteins. Some of the sequences with modified z-scores ranging from 1.5 to 3, derived from all the above proteins, were also analyzed. The known and speculated zDHHC17 interactors to be analyzed were picked from the zDHHC17 interactor list reviewed by Butland *et al.* in 2014 (11), and the most recently identified interactors and substrates of zDHHC17 (39, 53, 54). Additionally, proteins whose palmitoylation was decreased in zDHHC17-deficient mice (13), the known zDHHC13 substrate cornifelin (27), and related proteins were also included in the analysis. We first excluded sequences for which luminal, nuclear-only, mitochondrial, peroxisomal, and extracellular localization has been shown/proposed, as well as those that are predicted to be within transmembrane helices, or are located in regions previously crystallized (and are thus structured). For the remaining sequences in our list, we predicted disorder using either the DISOPRED3 tool (within the PSIPRED server; bioinf.cs.ucl.ac.uk/psipred/),^4^ or the PrDOS tool (Protein DisOrder Prediction; prdos.hgc.jp/cgi-bin/top.cgi),^4^ these two have been evaluated as the two most reliable disorder prediction tools, among the 28 prediction groups tested, by the recent Critical Assessment of techniques for protein Structure Prediction, CASP10 (55). From the sequences predicted to be cytosolic and disordered (at least 8 amino acids with a 10-amino acid stretch with Pro at position 7), 51 of the predicted top-scoring hits (modified z-scores above 6), and 56 other sequences with lower scores were synthesized as 12-mer peptides (positions 0–11) to be validated for ARzD17 binding.

### SUS

5 μl of yeast matings expressing both zDHHC-Cub-PLV bait and NubG-2HA-tagged prey at corresponding optic densities (*A*_600_) were dropped on synthetic defined (SD) media, to assess interactions, and on SD media supplemented with adenine and histidine to verify equal optical density among matings. The SUS principle is briefly described in Fig. 5*B,* legend; the procedures for yeast transformation, selection of plasmids, yeast-mating, as well as yeast media formulations have been described before (7, 56).

### Pulldown assays

For ARzD17-His pull downs, HEK293T cells expressing the corresponding EGFP-tagged proteins in 6-well plates were lysed by addition of 600 μl of lysis buffer (20 mm Tris-HCl, pH 8, 150 mm NaCl, 1% Triton X-100, 20 mm imidazole); 200 μl of the corresponding lysate was then diluted in 1 ml with lysis buffer without Triton, and diluted lysates were incubated with 25 μl of nickel-nitrilotriacetic acid-agarose resin (Qiagen, Manchester, UK), and 31 μl (50 μg) of ARzD17-His (or equivalent volume of ARzD17-His buffer) for 2 h at 4 °C. After extensive washing with washing buffer (20 mm Tris-HCl, pH 8, 300 mm NaCl, 1% Triton, 40 mm imidazole), bound proteins were eluted by boiling in 50 μl of Laemmli sample buffer. 7.5% of total inputs and 40% of total bound fractions were run on 12% SDS-polyacrylamide gels, and following electrophoresis and transfer to nitrocellulose, bound ARzD17-His was detected by Ponceau S staining and EGFP-tagged proteins by Western blotting using a GFP antibody.

### Co-immunoprecipitations

For co-immunoprecipitation assays, HEK293T cells in 24-well plates were co-transfected with murine zDHHC17 in HA-pEF-BOS plasmid (or empty vector) and EGFP, EGFP-PAI-RBP1, or EGFP-SLAIN1 plasmids in triplicate wells. Following 24 h, cells in each well were lysed in 100 μl of lysis buffer (Miltenyi Biotech) and lysates from three identical wells were pooled for co-immunoprecipitation assays. Protein isolation was performed using μMACS HA Isolation kit (Miltenyi Biotec), according to the manufacturer's protocol; however, to preserve binding of proteins to HA-zDHHC17, all washes were performed in lysis buffer.

## Author contributions

K. L. and L. H. C. designed the experiments. L. H. C. performed the immunoprecipitation experiments described in the legend to Fig. 7*C*. K. L. performed all other experiments and subsequent analyses. R. M. and G. B. constructed the peptide arrays. K. L. and L. H. C. wrote the manuscript. All authors approved the final version of the manuscript.

## Supplementary Material

Supplemental Data

## Footnotes

4 Please note that the JBC is not responsible for the long-term archiving and maintenance of this site or any other third party hosted site.

## Footnotes

3 The abbreviations used are:

AR: ankyrin repeat
PSSM: position-specific scoring matrix
MGAP: MAX gene-associated protein
CSPα: cysteine string protein α
SPR: Sprouty
SUS: split-ubiquitin system
EVL: Ena/VASP-like
PAI-RB1: plasminogen activator inhibitor 1 RNA-binding protein
zDABM: zDHHC-AR-binding motif
EGFP: enhanced green fluorescent protein.
