# Supplementary material for: Peptide array-based screening reveals a large number of proteins interacting with the ankyrin-repeat domain of the zDHHC17 S-acyltransferase
Source: J Biol Chem. 2017 Sep 7;292(42):17190–202. doi: 10.1074/jbc.M117.799650 (PMC5655499; doi:10.1074/jbc.M117.799650)
Supplement: Supplemental Data [file supp_292_42_17190__index.html]

Peptide array based screening reveals a large number of proteins interacting with the ankyrin repeat domain of the zDHHC17 S-acyltransferase — Peptide array-based screening reveals a large number of proteins interacting with the ankyrin-repeat domain of the zDHHC17 S-acyltransferase — Prediction and validation of zDHHC17-interacting proteins — Supplemental Data 

# Peptide array-based screening reveals a large number of proteins interacting with the ankyrin-repeat domain of the zDHHC17 *S*-acyltransferase

## Supplemental Data

- supplemental data and figure S1 (.pdf, 556 KB) - Position Specific Scoring Matrices (PSSMs) produced from quantification of spots in SNAP25 and CSP peptide-derived arrays.
- supplementary tables S1-3 (.xlsx, 30.7 MB) - Table S1. Peptides scored in Scansite 3 matching input motif. Table S2. Peptides scored in Scansite 3 matching input motif and additionally predicted to be disordered and cytosolic. Table S3. Prediction of palmitoylation sites among validated zDABM-containing proteins, using CSS-Palm 3.0 (high stringency setting).
