# Supplementary material for: Peptide array-based screening reveals a large number of proteins interacting with the ankyrin-repeat domain of the zDHHC17 S-acyltransferase
Source: J Biol Chem. 2017 Sep 7;292(42):17190–202. doi: 10.1074/jbc.M117.799650 (PMC5655499; doi:10.1074/jbc.M117.799650)
Supplement: Supplemental Data [file 10.1074_M117.799650_jbc.M117.799650-1.pdf]

Peptide array–based screening reveals a large number of proteins interacting with the ankyrin-repeat domain of the zDHHC17 S-acyltransferase

Kimon Lemonidis, Ruth MacLeod, George S. Baillie, Luke H. Chamberlain

Supplemental Data

Figure S1. Position Specific Scoring Matrices (PSSMs) produced from quantification of spots in SNAP25 and CSP peptide-derived arrays.

Table S1. Peptides scored in Scansite 3 matching input motif.

Table S2. Peptides scored in Scansite 3 matching input motif and additionally predicted to be disordered and cytosolic.

Table S3. Prediction of palmitoylation sites among validated zDABM-containing proteins, using CSS-Palm 3.0 (high stringency setting).

A

SNAP25

Amino acid substitution	Position 1	2	3	4	5	6	7	8	9	10
A	0.0422	0.0000	0.0000	0.0326	0.0385	0.0000	0.0000	0.0487	0.0502	0.0586
R	0.0851	0.0000	0.0231	0.1067	0.1459	0.0000	0.0000	0.1556	0.4796	0.1992
N	0.0422	0.0000	0.0000	0.0337	0.0388	0.0000	0.0000	0.0237	0.0000	0.0261
D	0.0231	0.0000	0.0000	0.0234	0.0087	0.0000	0.0000	0.0000	0.0000	0.0031
C	0.0777	0.0920	0.0623	0.0389	0.0554	0.4254	0.0000	0.0632	0.0667	0.0318
Q	0.0506	0.0000	0.0000	0.0442	0.0476	0.4816	0.0000	0.0623	0.0000	0.0316
E	0.0314	0.0000	0.0000	0.0185	0.0221	0.0000	0.0000	0.0134	0.0000	0.0047
G	0.0571	0.0000	0.0000	0.0049	0.0000	0.0000	0.0000	0.0960	0.0487	0.0253
H	0.0825	0.0000	0.0000	0.0407	0.0639	0.0000	0.0000	0.0535	0.0331	0.0560
I	0.0693	0.5640	0.6799	0.1091	0.0899	0.0000	0.0519	0.0339	0.0000	0.0389
L	0.0417	0.0000	0.0000	0.1033	0.0539	0.0000	0.0000	0.0455	0.0000	0.0376
K	0.0616	0.0000	0.0000	0.0700	0.1318	0.0000	0.0000	0.1210	0.2309	0.0503
M	0.0425	0.0000	0.0000	0.0335	0.0598	0.0931	0.0000	0.0676	0.0000	0.0354
F	0.0621	0.0000	0.0402	0.0560	0.0398	0.0000	0.0000	0.0507	0.0000	0.0799
P	0.0372	0.0511	0.0000	0.0000	0.0000	0.0000	0.9481	0.0097	0.0450	0.0053
S	0.0271	0.0000	0.0000	0.0386	0.0413	0.0000	0.0000	0.0223	0.0242	0.0410
T	0.0401	0.0000	0.0000	0.1053	0.0510	0.0000	0.0000	0.0287	0.0000	0.0329
W	0.0655	0.0000	0.0000	0.0574	0.0335	0.0000	0.0000	0.0464	0.0216	0.1325
Y	0.0338	0.0000	0.0000	0.0276	0.0244	0.0000	0.0000	0.0355	0.0000	0.0877
V	0.0271	0.2930	0.1945	0.0555	0.0535	0.0000	0.0000	0.0223	0.0000	0.0220

CSPα

Amino acid substitution	Position 1	2	3	4	5	6	7	8	9	10
A	0.0186	0.1153	0.0000	0.0000	0.0180	0.0000	0.0000	0.0299	0.0616	0.0358
R	0.0335	0.0000	0.0000	0.0723	0.0472	0.0000	0.0000	0.0764	0.1448	0.1108
N	0.0320	0.0000	0.0000	0.0145	0.0359	0.0000	0.0000	0.0328	0.0299	0.0556
D	0.0257	0.0000	0.0000	0.0000	0.0292	0.0000	0.0000	0.0000	0.0000	0.0110
C	0.0389	0.0501	0.0362	0.0673	0.0669	0.4109	0.0000	0.0352	0.0563	0.0411
Q	0.0568	0.0000	0.0000	0.0560	0.0581	0.4250	0.0000	0.0902	0.0563	0.0900
E	0.0519	0.0203	0.0000	0.0085	0.0345	0.0000	0.0000	0.0136	0.0000	0.0190
G	0.0675	0.0213	0.0000	0.0000	0.0097	0.0000	0.0000	0.0896	0.0757	0.0472
H	0.0693	0.0460	0.0000	0.0430	0.0733	0.0000	0.0000	0.0543	0.0514	0.0664
I	0.0771	0.0485	0.6597	0.1308	0.1090	0.0000	0.0000	0.0547	0.0567	0.0909
L	0.0856	0.0000	0.0000	0.1070	0.0782	0.0000	0.0000	0.0699	0.0303	0.0671
K	0.0798	0.0000	0.0000	0.0476	0.0942	0.0000	0.0000	0.0838	0.1026	0.0313
M	0.0633	0.0000	0.0000	0.0389	0.0750	0.1641	0.0000	0.0730	0.0461	0.0543
F	0.0776	0.0000	0.0000	0.0664	0.0680	0.0000	0.0000	0.1043	0.0678	0.0605
P	0.0595	0.3573	0.0000	0.0000	0.0000	0.0000	1.0000	0.0189	0.0665	0.0187
S	0.0331	0.0000	0.0000	0.0681	0.0639	0.0000	0.0000	0.0361	0.0452	0.0245
T	0.0297	0.0502	0.0000	0.1379	0.0583	0.0000	0.0000	0.0400	0.0239	0.0437
W	0.0326	0.0254	0.0000	0.0526	0.0273	0.0000	0.0000	0.0404	0.0465	0.0792
Y	0.0411	0.0000	0.0000	0.0097	0.0164	0.0000	0.0000	0.0359	0.0271	0.0362
V	0.0262	0.2657	0.3041	0.0794	0.0370	0.0000	0.0000	0.0211	0.0112	0.0168

B

Position	Amino acid substitution A	R	N	D	C	Q	E	G	H	I	L	K	M	F	P	S	T	W	Y	V
0	1	1	1	1	1	1	1	1	1	1	1	1	1	1	1	1	1	1	1	1
1	0.61	1.19	0.74	0.49	1.17	1.07	0.83	1.25	1.52	1.46	1.27	1.41	1.06	1.4	0.97	0.6	0.7	0.98	0.75	0.53
2	1.18	0.05	0.05	0.05	1.42	0.05	0.23	0.24	0.48	6.12	0.05	0.05	0.05	0.05	4.08	0.05	0.53	0.28	0.05	5.59
3	0.05	0.26	0.05	0.05	0.98	0.05	0.05	0.05	0.05	13.4	0.05	0.05	0.05	0.43	0.05	0.05	0.05	0.05	0.05	4.99
4	0.35	1.79	0.48	0.26	1.06	1	0.27	0.07	0.84	2.4	2.1	1.18	0.72	1.22	0.05	1.07	2.43	1.1	0.37	1.35
5	0.57	1.93	0.75	0.38	1.22	1.06	0.57	0.12	1.37	1.99	1.32	2.26	1.35	1.08	0.05	1.05	1.09	0.61	0.41	0.91
6	0.05	0.05	0.05	0.05	8.36	9.07	0.05	0.05	0.05	0.05	0.05	0.05	2.57	0.05	0.05	0.05	0.05	0.05	0.05	0.05
7	0	0	0	0	0	0	0	0	0	0	0	0	0	0	21	0	0	0	0	0
8	0.79	2.32	0.56	0.05	0.98	1.53	0.27	1.86	1.08	0.89	1.15	2.05	1.41	1.55	0.29	0.58	0.69	0.87	0.71	0.43
9	1.12	6.24	0.32	0.05	1.23	0.59	0.05	1.24	0.85	0.59	0.33	3.33	0.49	0.7	1.12	0.69	0.26	0.68	0.3	0.14
10	0.94	3.1	0.82	0.14	0.73	1.22	0.24	0.72	1.22	1.3	1.05	0.82	0.9	1.4	0.24	0.65	0.77	2.12	1.24	0.39
11	1	1	1	1	1	1	1	1	1	1	1	1	1	1	1	1	1	1	1	1
12	1	1	1	1	1	1	1	1	1	1	1	1	1	1	1	1	1	1	1	1
13	1	1	1	1	1	1	1	1	1	1	1	1	1	1	1	1	1	1	1	1
14	1	1	1	1	1	1	1	1	1	1	1	1	1	1	1	1	1	1	1	1

Figure S1. Position Specific Scoring Matrices (PSSMs) produced from quantification of spots in SNAP25 and CSP peptide-derived arrays. A. SNAP25 and CSP PSSMs used for generation of Sequence logos. Peptides whose signal intensities were below 5% of average intensity value of wild-type peptides were penalised with a value of zero (shown in red). **B.** PSSM used as input motif for the search of human sequences matching this motif in Scansite 3. Positions for which no binding data is available are given a score of 1 (no preference). Strongly disfavorable substitutions (scores of 0.05 and below) are shown in red.
